# Case report: a 3-year follow-up on nodal marginal zone lymphoma coexisting with disseminated Talaromyces marneffei infection in a non-endemic area

**DOI:** 10.3389/fonc.2024.1407893

**Published:** 2025-01-06

**Authors:** Xiping Yu, Houyun Xu, Junjie Zhou, Zhiqiang Zhu, Baizhou Li

**Affiliations:** ^1^ Department of Pathology, The Fourth Affiliated Hospital of School of Medicine, and International School of Medicine, International Institutes of Medicine, Zhejiang University, Zhejiang, Yiwu, China; ^2^ Department of Radiology, The Fourth Affiliated Hospital of School of Medicine, and International School of Medicine, International Institutes of Medicine, Zhejiang University, Zhejiang, Yiwu, China; ^3^ Department of Clinical Laboratory, The Fourth Affiliated Hospital of School of Medicine, and International School of Medicine, International Institutes of Medicine, Zhejiang University, Zhejiang, Yiwu, China

**Keywords:** *Talaromyces marneffei*, nodal marginal zone lymphoma, infection, diagnosis, treatment

## Abstract

This case report presents the details of an elderly man in Zhejiang Province of China, who tested human immunodeficiency virus (HIV) negative and subsequently developed Nodal Marginal Zone Lymphoma (NMZL) along with *T. marneffei* disseminated infection. The study focuses on analyzing the distinct clinical symptoms and pathological manifestations in order to offer precise diagnosis and effective treatment for patients. A 76-year-old male patient was admitted to our hospital due to recurrent fever. An enhanced CT of the abdomen revealed bilateral renal and adrenal mass, along with multiple enlarged lymph nodes in the retroperitoneum. A chest un-enhanced CT showed flaky high-density shadows in the left lung, along with multiple enlarged lymph nodes in the lung hilar, mediastinal regions, and the left supraclavicular side. Subsequently, we conducted a biopsy of the left supraclavicular lymph node and confirmed the NMZL combined with *T. marneffei* infection. Over 3 years follow-up, there was no recurrence of *T. marneffei* infection and the lymphoma remained stable. Clinicians and pathologists should remain vigilant about the possibility of *T.marneffei* infection in non-prone areas. When encountering patients with unexplained fever, swollen lymph nodes, and multiple systems involvement, it is crucial to consider the possibility of lymphoma and fungal infections coexisting.

## Introduction

Talaromyces marneffei, a rare opportunistic pathogen, is known for causing disseminated and potentially fatal infections, particularly in subtropical regions. It is predominantly observed in individuals with compromised immune systems, such as those with AIDS and other immunocompromised conditions. However, individuals without AIDS but with underlying diseases may also be susceptible to *T. marneffei* when faced with severe systemic infections (1, 2). Clinical manifestations of *T. marneffei* include fever, cough, sputum production, lymphadenopathy, and hepatosplenomegaly. Notably, the incidence of *T. marneffei* in non-AIDS patients has been on the rise in recent years (3). Despite this, there remains a significant gap in the understanding of the diagnosis and treatment of this disease among clinicians and pathologists. This study aims to broaden the knowledge base of this rare condition among medical professionals.

## Case description

**Figure 1:**
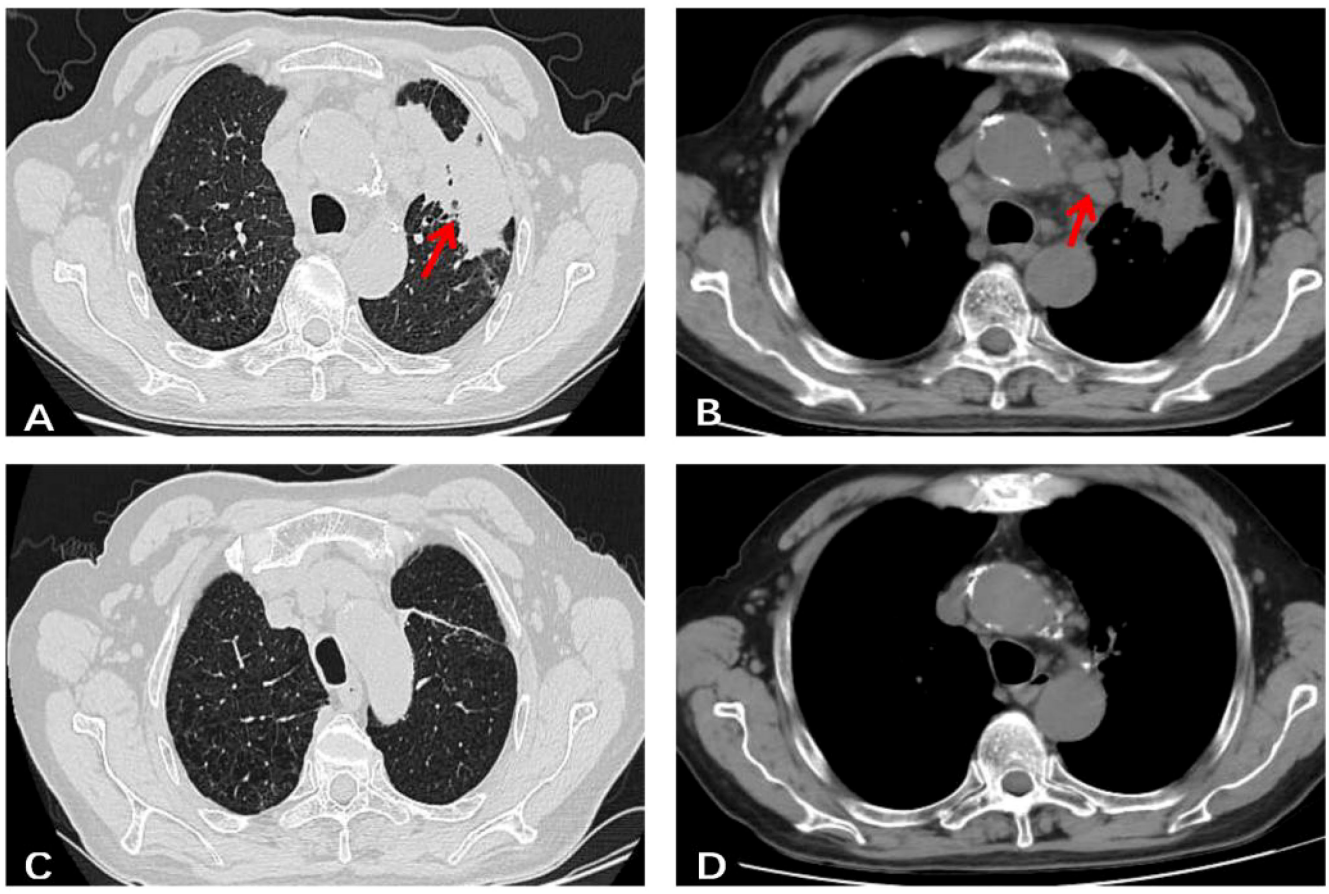
A chest un-enhanced CT of this case. **(A)** Patchy high-density shadows with poorly defined boundaries in the upper lobe of the left lung, as well as partial bronchial obstruction in the same area. **(B)** Multiple enlarged lymph nodes were observed in lung hilum and mediastinal region. **(C, D)** After standardized systemic treatment with oral voriconazole, pulmonary lesion gradually resolved, both of lung hilar and mediastinal region lymph nodes shrank obviously.

**Figure 2:**
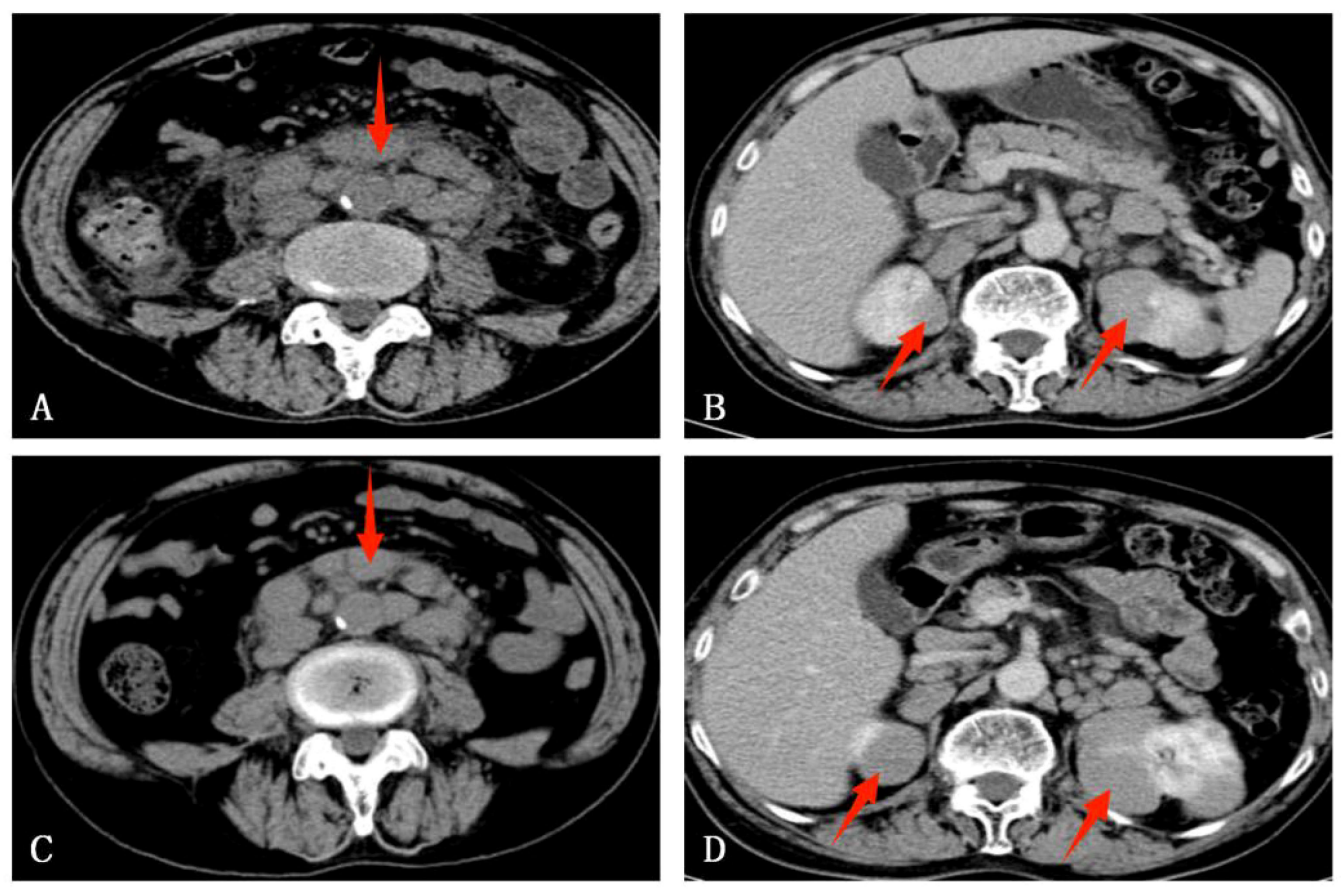
An abdominal enhanced CT of this case. **(A)** Multiple enlarged lymph nodes in the mesenteric and retroperitoneum region. **(B)** Bilateral adrenal glands and kidneys mass. **(C)** After standardized systemic treatment with oral voriconazole, there was no reduction in the size of the mesenteric and retroperitoneal lymph nodes. **(D)** Bilateral adrenal glands and renal masses seemingly enlarged.

A 76-year-old male, residing in Zhejiang Province, China, was hospitalized for a persistent fever and cough that had persisted for over 20 days. His medical history was unremarkable for hypertension, diabetes, cardiovascular disease, malignancies, tuberculosis, or other chronic infectious diseases. He had no record of travel to regions with disease outbreaks or contact with wild animals. The patient had a complete vaccination profile with positive serological responses to various vaccines, including Measles, Mumps, Rubella, Varicella, Pertussis, Tetanus, Polio, DPT, PPD, and Hepatitis B. There was no family history of congenital immunodeficiency. On initial examination, his maximum body temperature was recorded at 38.5°C. Laboratory investigations revealed elevated C-reactive protein (CRP) at 85.1mg/L, with a white blood cell count of 3.8×10^9^/L, lymphocyte count of 1.6×10^9^/L, lymphocyte percentage of 41.3%, red blood cell count of 2.87×10^12^/L, hemoglobin level of 92g/L, albumin level of 30.7g/L, and immunoglobulin level of 34.6g/L. The analysis of lymphocyte subsets in the peripheral blood revealed the following percentages: CD3+T cells constituted 64.49%, CD4+ T helper cells accounted for 42.68%, and CD8+ cytotoxic T cells made up 28.44%. CD19+B cells accounted for 16.32%, while natural killer (NK) cells marked by CD16 and CD56, comprised 17.61%. All lymphocyte counts fell within the normal range, with no detectable abnormalities noted. Urinalysis revealed the presence of red blood cells at 25/uL. Physical examination identified multiple lymphadenopathies in the left supraclavicular fossa and axillary regions, with the largest lymph node measuring 2.5×2cm. The abdominal examination was unremarkable for tenderness or skin lesions. Chest computed tomography (CT) demonstrated patchy high-density opacities in the upper lobe of the left lung and partial bronchial obstruction (**Figure 1A**). Enlarged lymph nodes were also noted in the lung hilum and mediastinal region (**Figure 1B**). Following a course of standardized systemic therapy with oral voriconazole, the pulmonary lesion showed a gradual resolution, and there was a noticeable reduction in the size of both the lung hilar and mediastinal lymph nodes, as depicted in **Figures 1C, D**. Abdominal contrast-enhanced CT revealed multiple enlarged lymph nodes in the mesenteric and retroperitoneal regions, along with masses in the bilateral adrenal glands and kidneys, as illustrated in **Figures 2A, B**. Despite undergoing standardized systemic treatment with oral voriconazole, there was no observed reduction in the size of the mesenteric and retroperitoneal lymph nodes, as shown in **Figure 2C**. Moreover, the masses in the bilateral adrenal glands and kidneys appeared to have enlarged, as seen in **Figure 2D**.

**Figure 3:**
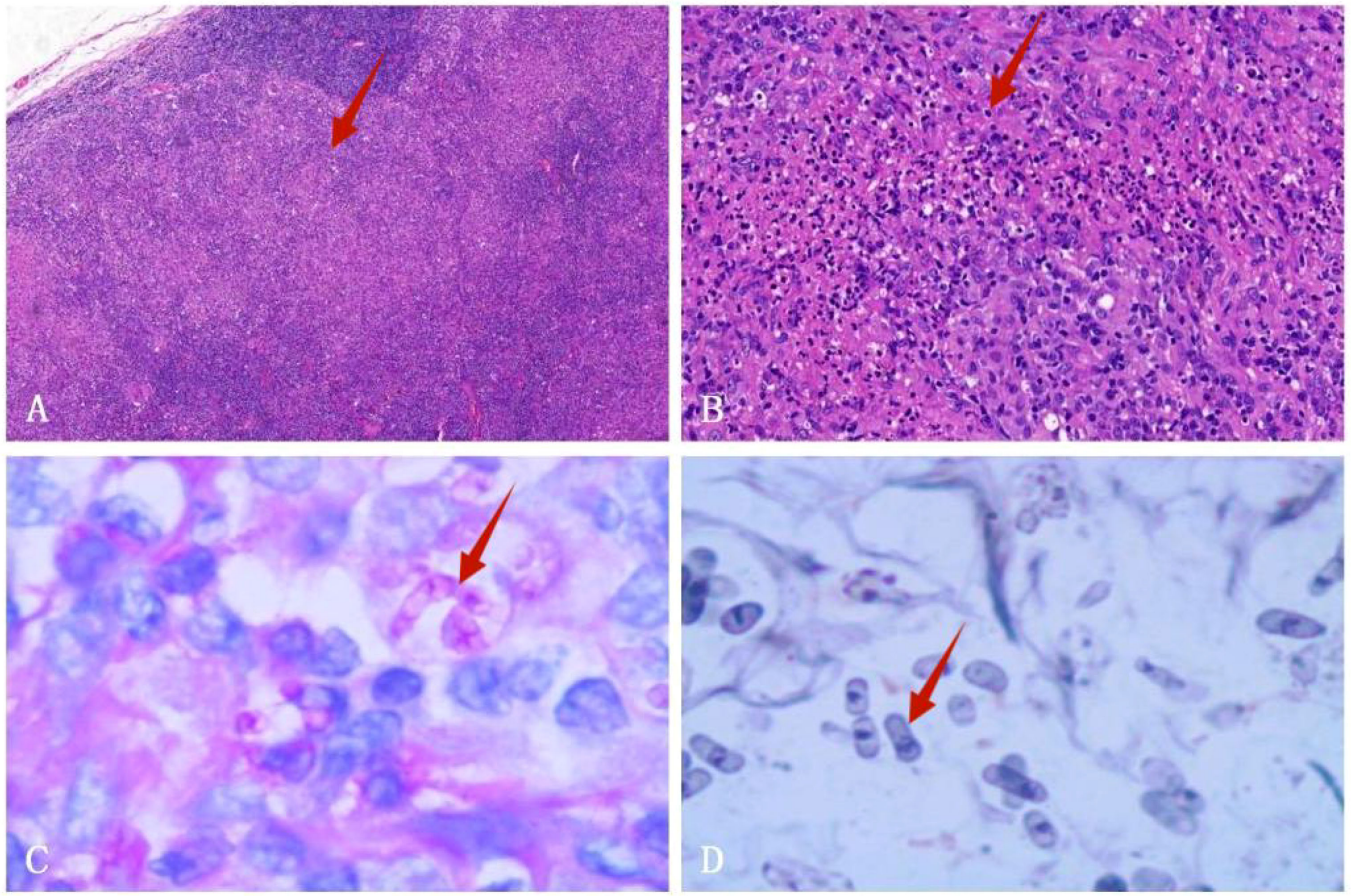
Left supraclavicular lymph node biopsy specimen. **(A)** HE staining shows fused granulomas that destroy the cortical area of the lymph nodes (HE×40). **(B)** HE staining displays a granulomatous structure with necrosis (HE×200). **(C)** PAS staining exhibits the presence of a transverse septum at the center of the long rod-shaped thallus (×1000 oil immersion lens). **(D)** Hexaammonium silver staining shows a transverse septum at the center of the long rod-shaped thallus (×1000 oil immersion lens).

Empirical anti-infective therapy was given, but the patient’s symptoms did not improve. On the fifth day after treatment, a fine needle aspiration biopsy of the left supraclavicular lymph node was performed. Pathology results showed structural destruction of the cortical area of the lymph node, with diffuse and well-defined granulomatous structures accompanied by necrosis (**Figures 3A, B**). Periodic Acid-Schiff (PAS) staining and hexaammonium silver staining were performed, revealing a large number of *T. marneffei* with transverse septum characteristics distributed in clusters (**Figures 3C, D**).

**Figure 4:**
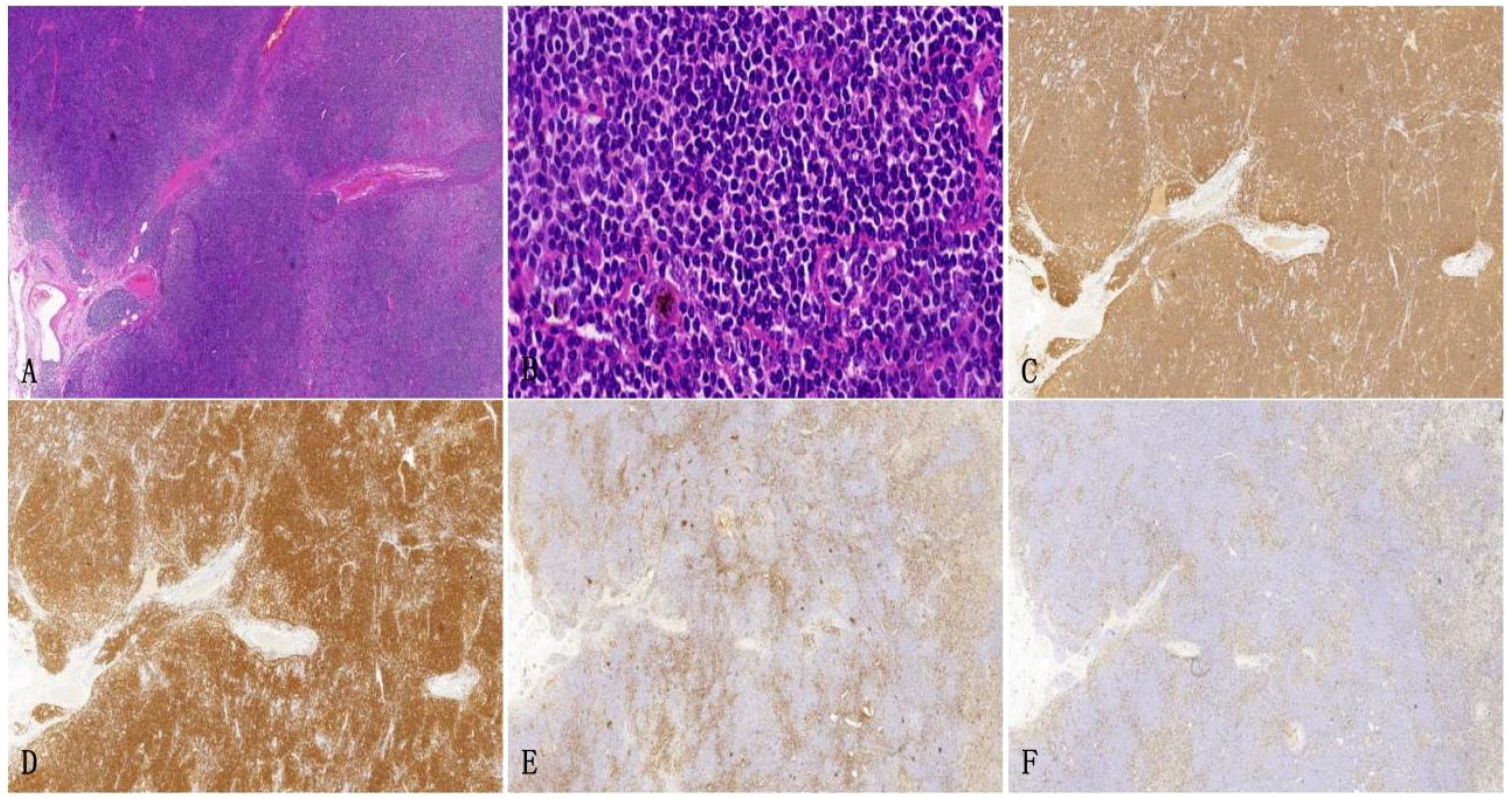
Left supraclavicular lymph node biopsy specimen. **(A)** HE staining depicts the growth of large diffuse tumorous small lymphoid cells that cause destruction in the medullary cord and medullary sinus area (HE×10). **(B)** HE staining reveals small lymphoid cells with irregular nuclei and slight chromatin clumps, nucleoli are not prominent and the cytoplasm appears transparent (× 200). **(C, D)** Immunohistochemical staining shows CD20 and CD79a are positive (× 100). **(E, F)** Immunohistochemical staining demonstrates the expression of KI-67 and CD3, outlining the nodular distribution of marginal zone lymphoma (×10).

**Figure 5:**
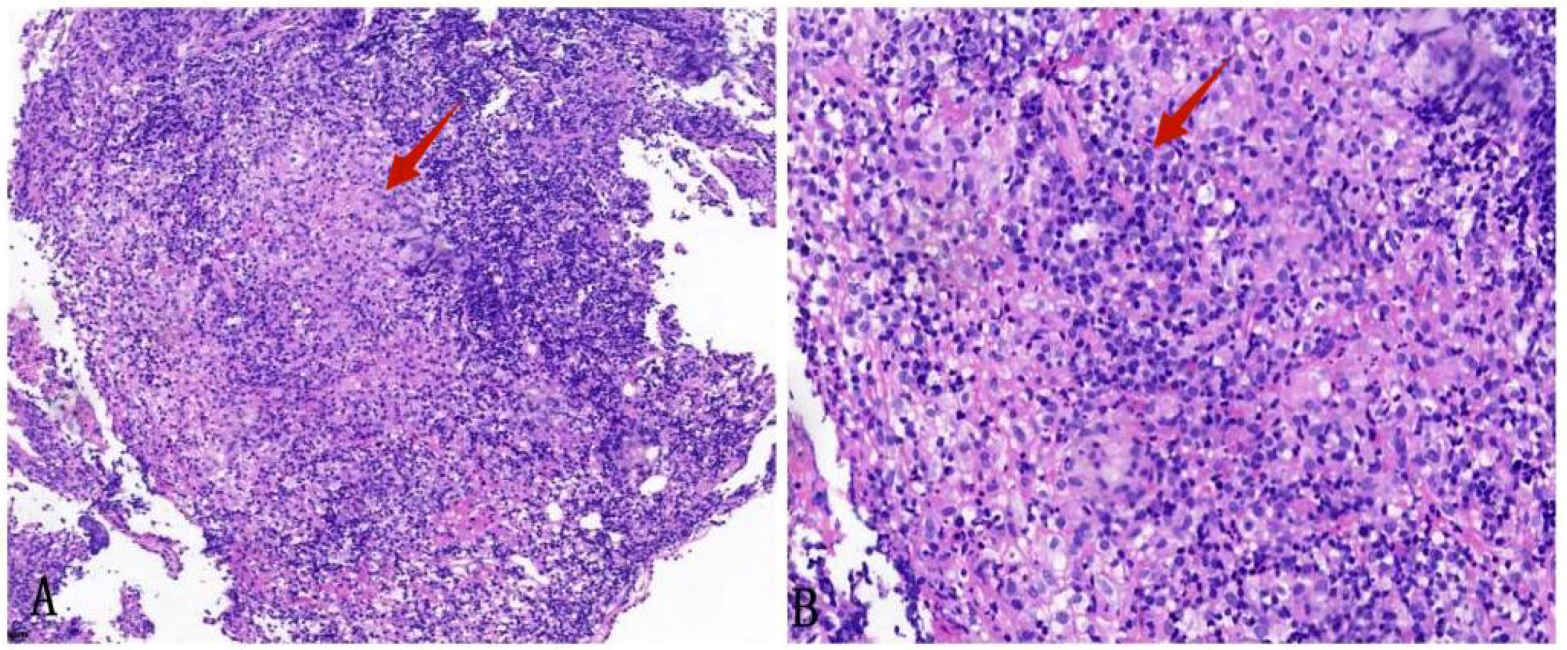
Biopsy specimen of the anterior segment of the left lung upper lobe. **(A)** HE staining shows chronic inflammation of the bronchial mucosa with loose granulomas (× 100). **(B)** HE staining shows a granulomatous structure with necrosis (× 200).

**Figure 6:**
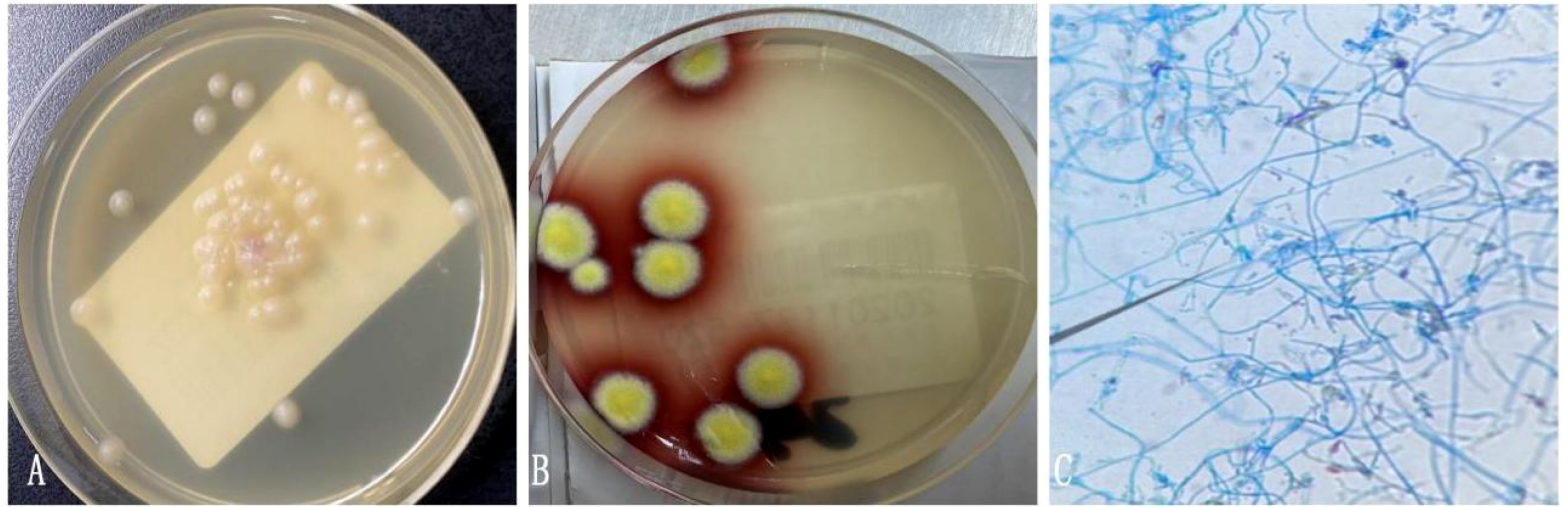
**(A)** Bronchial lavage fluid culture on CHROMagar Candida medium at 37°C showed yeast phase. **(B)** Bronchial lavage fluid culture of *T. marneffei* in the mold phase at 25°C, characteristic red pigment production was observed. **(C)** Lactophenol cotton blue staining from fungal Bronchial lavage fluid culture demonstrating *T. marneffei* bearing phialides and chains of conidia.

The architecture of the lymphatic cords and sinuses within the medullary region appeared indistinct, with a nodular pattern of small lymphoid cell infiltration noted in this area. Immunohistochemical analysis established the diagnosis of small B cell lymphoma, specifically NMZL (**Figure 4**), which was found to be co-infected with the disseminated pathogen *T. marneffei*. To rule out a pulmonary neoplasm, a fiberoptic bronchoscopy was conducted. The procedure revealed obstructive white necrotic material at the orifice of the anterior segment of the left upper lobe and within the lumen of the right upper lobe. A biopsy was performed, and bronchial lavage fluid was obtained for microbial culture. The bronchial biopsy indicated chronic granulomatous inflammation with necrosis (**Figure 5**). Stains using Periodic Acid-Schiff (PAS) and hexaamine silver were negative for fungal elements. Nonetheless, the culture of the bronchial lavage fluid identified *T. marneffei*, which demonstrated hyaline, translucent, and septate hyphae at lower temperatures, transitioning to a yeast phase with oval-shaped thalli at higher temperatures (**Figure 6**). These findings confirmed the presence of pulmonary *T. marneffei* infection.

**Figure 7:**
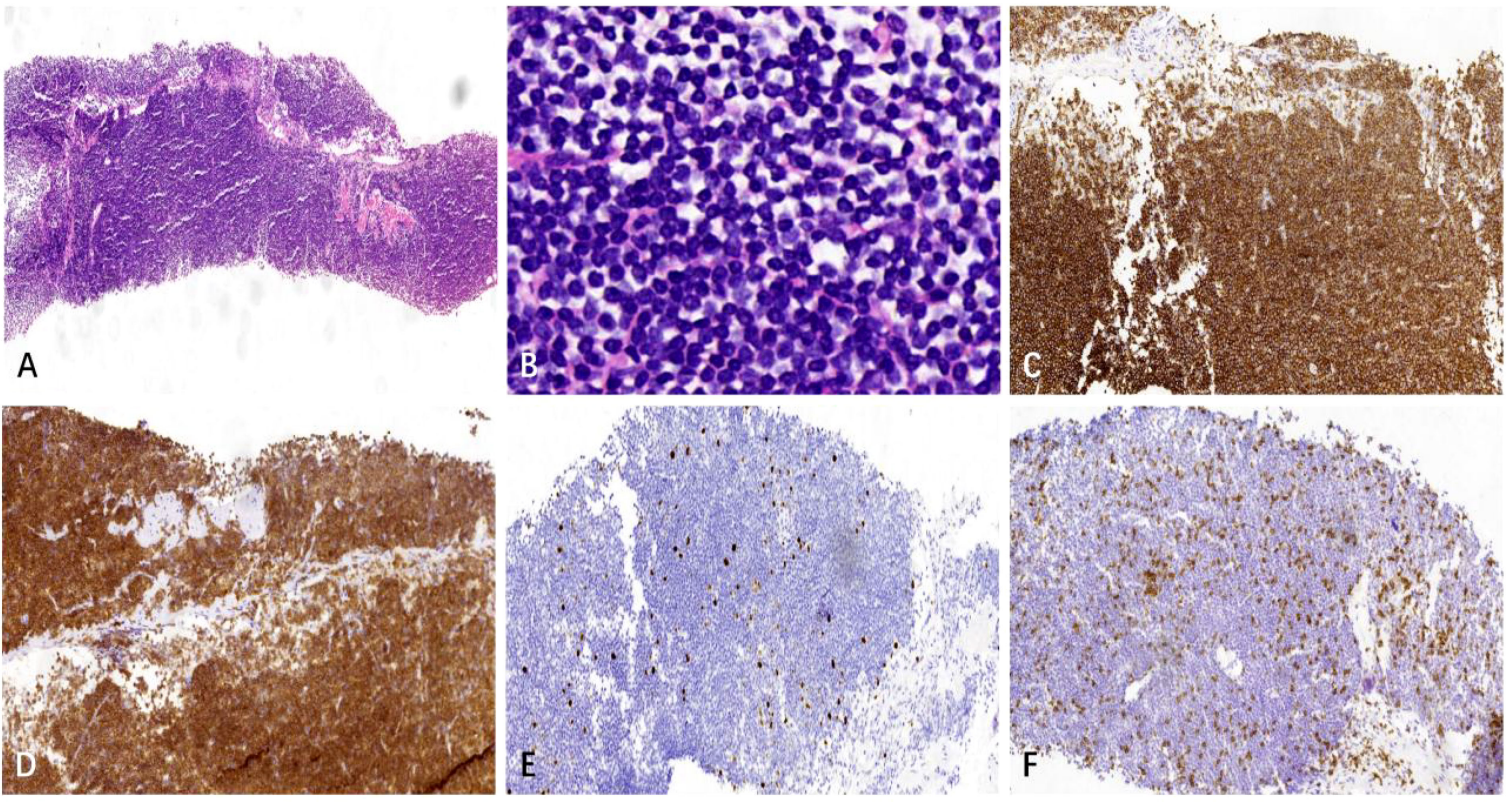
The left renal mass biopsy specimen. **(A)** HE staining shows diffuse growth of lymphoid cells in the punctured tissue (× 40). **(B)** HE staining shows small lymphoid cells with uniform size, round shape, fine chromatin, indistinct nucleoli, and little cytoplasm (× 400). **(C, D)** Immunohistochemical staining shows CD20 and CD79a are positive (× 100). **(E)** KI-67 indicates a relatively low tumor proliferation index(× 100), and **(F)** Immunohistochemical staining reveals a small number of scattered CD3+ T cells in the background(× 100).

**Figure 8:**
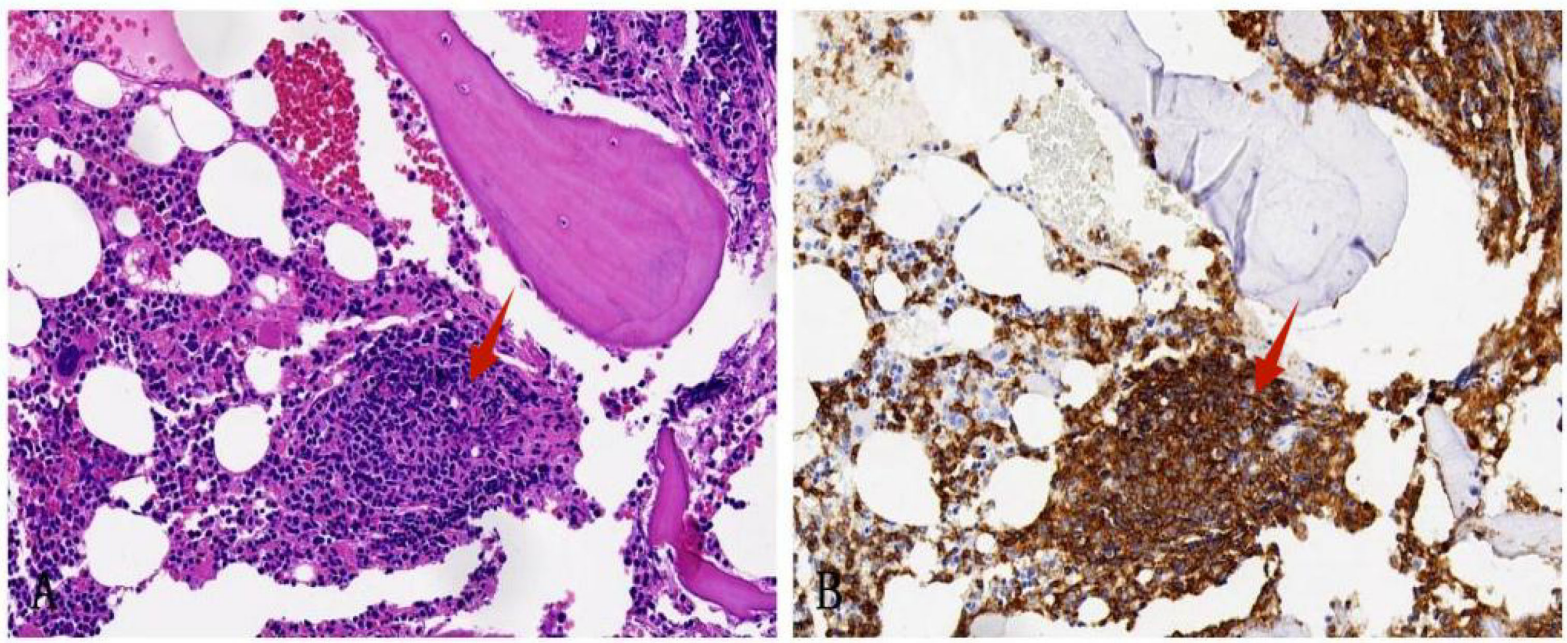
Bone marrow biopsy specimen. **(A)** HE staining shows the bone marrow is involved by nodal marginal zone lymphoma (×200); **(B)** Immunohistochemical staining shows that CD20 is positive (×200).

**Figure 9:**
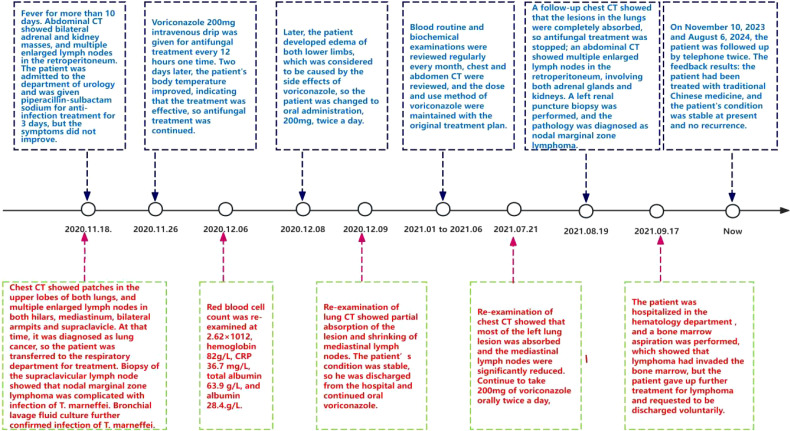
Showed the patient’s treatment process and response in detail.

Following a nine-month regimen of systemic therapy with oral voriconazole, there was a marked amelioration in the patient’s hematological parameters: CRP decreased to 2.2mg/L, hemoglobin level normalized to 126g/L, red blood cell count increased to 3.91×10^12^/L, albumin level rose to 42g/L, and immunoglobulin level improved to 41.1g/L. **Table 1** provides a comprehensive overview of the dosage, duration of treatment, efficacy indicators, and side effects associated with voriconazole throughout the treatment course. The patient exhibited a gradual resolution of pulmonary inflammation, accompanied by a notable reduction in the size of lymph nodes in the lung hilar and mediastinal regions. Despite this, the size of the mesenteric and retroperitoneal lymph nodes, as well as the bilateral adrenal glands and renal masses, remained unchanged post-treatment. Consequently, a biopsy of the left renal mass was conducted, revealing diffuse proliferation of small B cells upon HE staining. Immunohistochemical staining indicated low expression levels of KI-67 and CD3, aligning with the characteristics observed in the supraclavicular lymph node and confirming the presence of marginal zone lymphoma (**Figure 7**). Bone marrow aspiration further confirmed the involvement of the bone marrow by marginal zone lymphoma (**Figure 8**). A three-year follow-up revealed no recurrence of *T. marneffei* in the lungs, and the patient’s lymphoma remained stable. To elucidate the chronological progression and interrelationships of the patient’s condition, we have compiled all pertinent information in a logical, timeline-based manner (**Figure 9**).

**Table 1:** Showed the dosage, treatment duration and efficacy of voriconazole.

Drug name	Dosage	Usage	Side-effect	Usage change	Imaging evaluation	laboratory evaluation	Follow-up	Outcome
Voriconazole	200mg	Intravenous injection,once every 12 hours (2020.11.26)	After 15 days of treatment, oedema of both lower limbs developed(2020.12.08)	Because of Side-effect, Change to oral,200mg,twice one day(2020.12.08)	CT indicated pulmonary lesion partial absorption,which suggested the anti-fungal therapy was effective (2020.12.09)	Leucocyte count 6.7×10^9^, red-cell count 3.42×10^12^, hemoglobin 112g/L, C- reactive protein 3.4mg/L, Total albumin 88.2g/L, albumin 43.9g/L (2020.12.25)	In all follow-up for 10 times, mainly check blood routine, biochemical and chest CT. CT showed complete absorption of pulmonary lesions, mediastinal lymph nodes shrinked. All the time, oral voriconazole, 200mg, twice a day (duration of treatment a total of nearly 9 months).	Last follow-up revealed, leucocyte count 4.5×10^9^, red-cell count 3.45×10^12^, hemoglobin 116g/L, C reactive protein 3.2mg/L, Total albumin 88.7g/L, albumin 44.2g/L. Telephone-follow-up showed the patient’s condition remained stable and no recurrence.

## Discussion

*T. marneffei*, a rare opportunistic pathogen, is notorious for causing life-threatening disseminated infections, particularly in subtropical regions. It predominantly affects individuals with compromised immunity, such as those with AIDS and other immunocompromising conditions. However, the susceptibility to *T. marneffei* extends to individuals without AIDS who have underlying diseases, especially when they experience severe systemic infections (1, 2). The hallmark clinical features of *T. marneffei* infection encompass fever, cough, sputum production, and lymphadenopathy. Notably, there has been a consistent rise in the prevalence of *T. marneffei* in non-AIDS patients in recent years (3). According to report, exposure to agricultural soil constitutes a significant risk factor, with humidity emerging as the most critical environmental predictor for *T. marneffei* infection. Epidemiological data suggest potential transmission through the inhalation of *T. marneffei* conidia present in soil, with the skin or digestive tract also serving as possible portals of entry. Among HIV-negative patients with *T. marneffei*, farmers are disproportionately affected, with a higher incidence in males, likely due to their predominant role in agricultural and outdoor labor, which increases their exposure to the pathogen. Additionally, HIV-negative patients with *T. marneffei* tend to be significantly older than their HIV-positive counterparts, indicating that age may be a contributing risk factor. Despite these insights, there remains a considerable gap in the diagnostic and therapeutic understanding of this disease among clinicians and pathologists. Thus, the aim of this study is to augment the knowledge base of medical professionals regarding this uncommon but significant disease.

He L, et al. (4) reviewed the characteristics and clinical features of *T. marneffei* infection in non-HIV-infected patients in mainland China from 2001 to 2019, A total of 162 cases were reported, who were from 16 provinces of Mainland China, mainly higher prevalence in Guangxi Province and Guangdong Province. The infection showed a sex bias with a ratio of 2.84:1, with mean age of 37.0 years. There were 51 cases (31.48%) with underlying disease and 111 cases (68.52%) without any underlying disease, including diabetes(15.69%), tumor (13.73%), kidney transplantation(13.73%),tuberculosis(13.73%), SLE(7.84%) and so on. Therefore, immunological examination and genetic testing should be performed in HIV negative patients with *T. marneffei* infection. Laboratory manifestations of patients with *T. marneffei* infection often appear hematological abnormality—hypohemoglobinemia. In addition, significant increases in temperature and serum levels of CRP indicated fungal activity. Therefore, low hemoglobin may be a characteristic laboratory manifestations in non-AIDS patients with *T. marneffei* infection, which is useful for making early diagnosis. Microbiological culture from a variety of specimens, including blood, skin, bone marrow, lymph nodes, respiratory sources and so on, remains the gold standard for the diagnosis of *T. marneffei*. The diagnosis efficiency of *T. marneffei* infection by fungal culture has been reported with 100%, 90% and 76% sensitivity from bone marrow, skin biopsy and blood, respectively. Sometimes imaging methods(US,CT, MRI, and PET-CT) can provide additional information for diagnosis and treatment of *T. marneffei* infection. There are other available methods that can be helpful for the diagnosis of *T. marneffei*. For example, Metagenomenext-generation sequencing, (mNGS) can quickly test tens of thousands of bacteria, fungi, viruses, parasites, and mycobacteria, greatly expanded the pathogen spectrum. The mNGS is very suitable for immunocompromised patients with clinically suspected infection. Compared with the traditional methods, the actual detection time of mNGS was only 24 hours, which significantly shortened the diagnosis time and greatly improved the diagnostic efficiency. The diagnostic sensitivity of mNGS was 91%, specificity was 89%. Therefore, mNGS may be an important tool to accelerate diagnosis, especially in complicated immunocompromised patients (5).In addition, in recent years, qPCR technology, as a non-culture method, has been widely used in the specific detection of various pathogenic fungi in clinical samples, which have shown good diagnostic efficacy.

Once diagnosed with *T. marneffei* infection, amphotericin B, itraconazole, fluconazole and voriconazole were regarded as first-line therapies. Current treatment guidelines recommend liposomal amphotericin B for two weeks, followed by oral itraconazole for ten weeks, followed by secondary prophylaxis. This regimen has resulted in a 97.3% cure rate in a non-randomized study (4). However, in our study the patient was mainly treated with the voriconazole, and finally the patient was completely cured. So we had summarized **Table 1** that showed detailedly the dosage, treatment duration, specific efficacy indicators and side-effect of voriconazole during the entire course of the treatment.

The exact pathogenic cause of *T. marneffei* fungal infection is currently unknown. It is believed that inhalation of *T. marneffei* fungal spores can lead to acute lung disease and can spread through the bloodstream to affect various organ systems such as the skin, reticuloendothelial system, bone marrow, and intestines (6–8). Phagocytic cells are the primary host defense against the fungus, resulting in granulomatous and suppurative reactions in immunocompetent patients and necrotizing reactions in immunocompromised patients. Previously, it was thought that *T. marneffei* infection was more common among HIV-positive patients. However, recent studies (9) have shown that HIV-negative patients with underlying diseases are also susceptible to *T. marneffei* infection. These underlying diseases mainly include connective tissue diseases, malignant tumors, diabetes, and organ transplantation (2). However, the case of *T. marneffei* fungal infection combined with NMZL is extremely rare. The patient’s primary symptoms included recurrent fever and cough, maximum body temperature 38.5°C. The chest un-enhanced CT revealed flake-like shadows in the upper lobes of both lungs, however, the patient’s pulmonary symptoms did not align with the imaging findings, which made clinical diagnosis more challenging. Additionally, painless enlargement of lymph nodes was observed in the left supraclavicular fossa. Abdominal enhanced CT detected multiple enlarged lymph nodes in the mesenteric and retroperitoneum area. Multiple masses were also observed in the bilateral kidney and adrenal gland. These imaging signs and clinical manifestations cannot be easily explained by *T. marneffei* fungal infection or lymphoma alone.

Post-biopsy histopathological analysis of the left supraclavicular lymph node demonstrated substantial cortical destruction with associated necrosis. Periodic Acid-Schiff (PAS) staining revealed the presence of purple-red fungal cells, crucial for differentiating *T. marneffei* from other fungal pathogens such as Histoplasma capsulatum and Cryptococcus. The distinct ‘sausage-shaped’ rod-like morphology and prominent septation are indicative of *T. marneffei*. The medullary region of the lymph node was severely compromised by the infiltration of small lymphoid tumor cells, leading to an indistinct medullary cord and sinus architecture. The tumor cells showed an indistinct nodular proliferation, a hallmark feature of NMZL. NMZL typically manifests as painless lymphadenopathy, often in the head and neck region (10),and may present with systemic symptoms like fever, night sweats, and weight loss. Bone marrow involvement is observed in 30%-40% of NMZL patients (11). NMZL arises from mature B cells within the lymph nodes and is categorized as one of the three subtypes of Marginal Zone Lymphoma (MZL), alongside extranodal marginal zone lymphoma (EMZL) and splenic marginal zone lymphoma (SMZL) (12). Accurate diagnosis of NMZL necessitates the exclusion of the other MZL subtypes. As noted by Wang L et al., monoclonal immunoglobulin (McIg) is a frequently secreted protein in plasmacytoma or B lymphocyte-associated lymphomas, presenting clinically as elevated levels of monoclonal immunoglobulin or its fragments in serum or urine, which is uncommon in NMZL (13). Economopoulos T et al. (14)analyzed the data of 255 B-NHL patients, McIg were negative in all 18 MZL patients. Wang F et al. (15)found that McIg-MZL is not clinically common. Upon thorough review of the patient’s family and medical history, no evidence of autoimmune-related diseases or significant proteinuria was found; serum globulin levels were mildly elevated but not markedly.

NMZL is a rare type of lymphoid tumor, with an annual incidence rate of less than 1 in 100,000. It accounts for only 1% to 2% of all lymphoid tumors (16). Additionally, NMZL represents approximately 10% of MZL, which is a lower percentage compared to all non-Hodgkin lymphomas (2% of NHL) (17, 18). The tumor cells of NMZL consist mainly of monocytic-like cells, centrocyte-like cells, small lymphoid cells, and blast-like cells. These cells may also exhibit plasmacytoid differentiation and occasionally exhibit Dutcher bodies. Typically, the tumor cells demonstrate a marginal zone-like/interfollicular pattern with a fuzzy nodular shape and diffuse sheet-like infiltrative growth. In advanced stages, the lymphatic structure was completely destroyed by the tumor cells, often infiltrating the remaining germinal centers and forming a “follicular implantation”. The tumor cells in this case primarily consist of small lymphoid cells, with a small number of monocyte-like cells, and grow in a fuzzy nodular shape. The majority of tumor cells that CD20 and CD79a are positive were found in the medullary area, leading to the destruction of the medullary cord structure in the medullary sinus. Immunohistochemistry for CD21 protein revealed a damaged follicular dendritic network, along with the presence of CD10 and BCL6 proteins, indicating “follicular implantation”. CD3 and Ki-67 stained immune proteins outline the shape of the nodule. T cells show more active proliferation in the inter-nodular area, leading to an increased Ki-67 proliferation index. However, the proliferation index of tumor cells within the nodule is not high, suggesting that is a low-risk grade lymphoma. The diagnosis of NMZL is determined by a process of exclusion. Histopathology alone is not sufficient for a definitive diagnosis. In NMZL, B-cell markers such as CD19, CD20, and CD79a are positive, which is the same as in other small B-cell lymphomas. However, specific immunohistochemical markers for B-cell lymphoma, including CD5, CD10, CD23, BCL6, CyclinD-1, and CD38, which are typically negative. These markers are helpful for excluding small lymphocytic lymphoma, follicular lymphoma, mantle cell lymphoma, and lymphoplasmacytic lymphoma. To obtain further clarity on the stage of lymphoma, the bone marrow aspiration revealed lymphoma involvement in the bone marrow.

Lymphoma patients have a weakened immune system, making them susceptible to viral, bacterial, and fungal infections. However, infection with *T. marneffei* is extremely rare (19). Although there is an increasing number of reported cases of *T. marneffei* infection, there is a lack of relevant genetic testing data, making it difficult to determine whether it is a primary or secondary infection. In our study, the patient initially presented with significant enlargement of the left supraclavicular lymph node, preceding the development of fever. We hypothesize that the patient was in the early stages of an indolent lymphoma that was subsequently complicated by *T. marneffei* infection. The initial symptoms, which included fever and generalized lymphadenopathy, closely resembled those of common infections. The concurrent presence of lymphoma and fungal infection further exacerbates the diagnostic complexity. Fortunately, the patient was promptly diagnosed with *T. marneffei* infection, and aggressive treatment led to successful management of the pulmonary infection. A follow-up three years post-discharge, conducted via telephone, revealed that the patient has been adhering to a regimen of traditional Chinese medicine and remains in a stable condition.

According to our investigation, the patient had no the family history of congenital immunodeficiency, and no MZL history in the past. It was the first discovered because of repeated fever, and antimicrobial therapy was ineffective. Then left supraclavicular lymph node and the left renal biopsy specimen conformed the MZL, So we considered the MZL may have no relationship to the inborn error of immunity. As the Sarah K et, al reported that adult immunodeficiency syndrome is strongly associated with neutralizing antibodies to interferon-γ, supporting the central role of interferon-γ in the control of numerous pathogens (20).The research findings show neutralizing anti-interferon-γ autoantibodies were detected in 88% of multiple opportunistic infections and were associated with an adult-onset immunodeficiency, like the advanced HIV infection. Regrettably, the patient didn’t perform the anti-interferon-γ antibodies and HLA testing. So we were not sure that the MZL was related to anti-interferon gamma antibodies, which is the limitation of our study. In the future we will pay more attention to this.

In conclusion, the main finding of this case is that patients who appear normal and do not have AIDS, organ transplantation, or other underlying diseases that impair immunity can still be infected with *T. marneffei*. It is important to thoroughly investigate whether the patient has other diseases such as lymphoma, solid tumors, or connective tissue disease. When encountering patients with unexplained fever, swollen lymph nodes, and multisystem involvement, any examinations and treatments don’t improved patients’ manifestations, it is necessary to consider the possibility of hematological tumors and fungal infections coexisting. Specifically, the coexistence of lymphoma and *T. marneffei* infections should be taken into account. The main significance of the cases is that contributes to the database of *T. marneffei* disease, demonstrating that it can also occur in non-endemic areas like Zhejiang, China. This challenges the notion that it is limited to subtropical regions. Therefore, clinicians and pathologists should remain vigilant about the possibility of *T. marneffei* infection in non-prone areas.

## Data Availability

The original contributions presented in the study are included in the article/supplementary material. Further inquiries can be directed to the corresponding author.
